# Bioactive Molecules from Extreme Environments II

**DOI:** 10.3390/md19110642

**Published:** 2021-11-17

**Authors:** Daniela Giordano

**Affiliations:** 1 Institute of Biosciences and BioResources (IBBR), CNR, Via Pietro Castellino 111, 80131 Napoli, Italy; daniela.giordano@ibbr.cnr.it; 2 Department of Marine Biotechnology, Stazione Zoologica Anton Dohrn (SZN), Villa Comunale, 80121 Napoli, Italy

Marine organisms are known to produce a wide variety of natural products that are unique in terms of diversity, structural, and functional properties [1].

Marine organisms living in extreme environments experience conditions close to the limit of life, e.g., polar and hot regions, deep sea, hydrothermal vents, marine areas of high pressure or high salinity. They have evolved unique strategies for surviving in these harsh conditions, as well as biosynthesizing novel bioactive compounds, which are potentially useful for pharmaceutical, cosmeceutical, nutraceutical, and biotechnological applications [2]. Research on extreme environments often requires complex and expensive infrastructure, as well as access. Recent technological developments have made these areas more accessible and, currently, research on extreme life is growing fast. However, the biodiversity in these hostile environments is still largely unknown, and it is expected that in the near future, further research will be dedicated to this field.

This Special Issue, as a continuation of the previous Special Issue, “Bioactive Molecules from Extreme Environments” (https://www.mdpi.com/journal/marinedrugs/special_issues/Extreme_Environments accessed on 4 November 2021), includes 10 research articles and 2 reviews, providing a wide overview of the chemical biodiversity offered by different marine organisms inhabiting extreme environments to be used for biotechnological and pharmaceutical applications. The six articles in this Special Issue are focused on the polar regions, which represent an untapped source of marine natural products and are still largely unexplored compared to more accessible sites. Many of these articles refer to Antarctica, which is the coldest and most inaccessible continent on the Earth, where extreme temperatures, light and ice have selected biological communities with a unique suite of bioactive metabolites. The marine organisms of Arctic and Antarctic environments are a reservoir of natural compounds, exhibiting huge structural diversity and significant bioactivities that could be used in human applications.

In Núñez-Pons et al. [3] authors firstly described the regulations on access and benefit sharing requirements for research in polar environments and then provided an overview of the molecules from Antarctic and Arctic marine organisms with promising biological activities. The main target of this review was to investigate bacteria and fungi as microbes and macroorganisms, such as cnidaria, bryozoa, mollusca, echinodermata, sponges, tunicates and macroalgae. The attention was focused on purified marine natural products with elucidated structures showing a biological activity against human pathogens. Terpenes, terpenoids and derivatives are the most commonly found compounds, whereas antimicrobial properties are the most widely reported activities from these environments. This survey was aimed at highlighting the chemical diversity of marine polar life and the versatility of a particular group of biomolecules with interesting biological activities.

Avila and Angulo-Preckler [4] wrote a comprehensive survey on bioactive compounds from heterobranch molluscs, which was very well documented by 876 references. In this paper, the authors analyzed the bioactivity of more than 450 compounds from ca. 400 species of heterobranch molluscs, many of which are from Antarctica. Molecules produced by molluscs, display ecological activities such as predator avoidance, toxicity, antifouling, trail-following and alarm pheromones, sunscreens, UV protection and tissue regeneration, whereas many other compounds display pharmacological activities. The most studied activities were cytotoxicity and anticancer and antibiotic activities.

As molluscs, sponges are known to produce a series of marine natural products with interesting biological activities that could be applied to biomedical applications. Riccio et al. [5] reported a bioassay-guided fractionation of four Antarctic sponges, *Mycale* (*Oxymycale*) *acerata, Haliclona* (*Rhizoniera*) *dancoi*, *Hemimycale topsenti* and *Hemigellius pilosus* that led to the identification of two different chemical classes of molecules, suberitenone A and B and mycalols. The fraction containing the marine mycalol and its analogues, which have already been reported to possess anticancer activity on anaplastic thyroid carcinoma cells [6,7], was identified as the most promising bioactive product. In fact, investigation at the gene and protein levels demonstrated that it may trigger ferroptosis in HepG2 cells.

The majority of marine bioactive compounds derive from microorganisms as an invaluable source for novel chemistry and sustainable production of bioactive compounds, bypassing the limit for the re-collection of marine resources using destructive practices [8]. However, many bioactive molecules obtained from host invertebrates are instead produced by their bacterial symbionts in the marine environment, highlighting their crucial roles in host-associated chemical defense [9]. In Sun et al. [10], spectroscopic and chemical analyses elucidated the structure of three new aspochracin-type cyclic tripeptides, sclerotiotides M–O, together with three known analogues, sclerotiotide L, sclerotiotide F and sclerotiotide B, obtained from the ethyl acetate extract of the fungus *Aspergillus insulicola* HDN151418, which was isolated from an unidentified Antarctic sponge. Sclerotiotides M and N showed antimicrobial activity against a panel of pathogenic strains. 

Di Lorenzo et al. [11] reported the structural characterization of the lipopolysaccharide’s glycolipid moiety, the lipid A, from three different psychrophilic bacteria belonging to the phylum of Proteobacteria isolated from Terra Nova Bay, Antarctica. Lipopolysaccharides are amphiphilic molecules exposed on the outer membrane of the Gram-negative bacteria that are essential for viability and survival. Under hostile conditions, lipopolysaccharides can undergo desaturation of the acyl chains, a reduction in length and an increase in their branching to provide further protection and facilitate adaptation. In cold-adapted bacteria, lipopolysaccharides display several uncommon structural features. Since lipid A is also involved in the innate immune response in mammals, the study of lipid A from cold-adapted bacteria is of great interest to understand the mechanism of cold adaptation for drug synthesis.

John et al. [12] described the production of copper nanoparticles using the green reduction of CuSO_4_ at low temperatures (22 °C) by using five Antarctic bacterial strains isolated from a consortium associated with the Antarctic ciliate *Euplotes focardii*. All copper nanoparticles display antimicrobial activity against various types of Gram-negative and Gram-positive bacteria and fungi pathogen microorganisms, including *Escherichia coli*, *Staphylococcus aureus*, and *Candida albicans*. The ability of these bacteria to synthesize copper nanoparticles may represent a mechanism of defense against this heavy metal. In fact, Antarctica is not completely free from contaminants that could reach the Southern Ocean via long-range atmospheric transport from other continents. Metal nanoparticle synthesis using microorganisms is an eco-friendly and sustainable strategy alternative to chemical and physical approaches, which is particularly promising in the bioremediation of the contaminated environment and in the production of antibiotics against various types of pathogenic microorganisms. Most research work in this field has been carried out on silver and gold nanoparticles. Another work on silver nanoparticles from Antarctic bacteria is given in the first book [13].

Du et al. [14] described the discovery and structural elucidation using spectroscopic analyses of two new secondary metabolites pyrrolidinone-bearing lipodipeptides, svalbamides A and B, identified in *Paenibacillus* sp. SVB7, which was isolated from the Svalbard archipelago in the Arctic Ocean. Svalbamides A and B are structurally unique as they contain 3-amino-2- pyrrolidinone amino acid, which is rarely reported in natural products, and 3-hydroxy-8-methyldecanoic acid was occasionally found in natural products from *Paenibacillus* and related bacteria. Svalbamides A and B may function as potential chemopreventive agents, as they induced quinone reductase activity in murine hepatoma cells.

In addition to the polar regions, extreme are also environments of deep sea, hydrothermal vents, hot/arid regions, etc and other articles review these environments in this Special Issue. 

Juhasz et al. [15] reported the structural elucidation of three dermacozines, dermacozines N–P, isolated from the piezotolerant actinomycete strain *Dermacoccus abyssi* MT 1.1^T^, from a Mariana Trench sediment in 2006, which was collected at a depth of 10,898 m from the Challenger Deep by the remotely operated submersible Kaiko in 1998. In the past, seven highly colored dermacozines A–G and 4 derivatives dermacozines H–J were isolated from this promising strain [16,17,18]. Dermacozine N is unique among phenoxazines because it bears a novel linear pentacyclic phenoxazine framework, is never reported as a natural product, and displays near-infrared absorption maxima, making it an excellent candidate for research in biosensing chemistry, photodynamic therapy, and opto-electronic applications. Moreover, dermacozine N possesses weak cytotoxic activity against melanoma (A2058) and hepatocellular carcinoma cells (HepG2), with IC50 values of 51 and 38 mM, respectively.

Singh and et al. [19] described *Bacillus amyloliquefaciens,* a thermotolerant marine strain S185 isolated from offshore the South Sea, China. This marine strain displayed a strong antifungal activity against *Fusarium oxysporum* f. sp. cubense (Foc), which is responsible for a severe fungal disease in banana plants (Panama disease), due to its capacity to produce the antifungal compound iturin A5. This strain is able to grow between 20–50 °C, adapting to variable conditions of pH, salts and temperature. Due to these features, it is a promising candidate for a cost-effective and sustainable biocontrol application for Panama disease in the future. 

Xing et al. [20] reported the isolation, structure elucidation, and biological activity of 10 new and 26 known compounds isolated from *Penicillium griseofulvum* MCCC 3A00225, a deep sea-derived fungus isolated from the Indian Ocean sediment. The *Penicillium* species is recognized as the richest source for biologically important and structurally unique secondary metabolites. All isolates were tested for in vitro anti-food allergic bioactivities in immunoglobulin (Ig) E-mediated rat basophilic leukemia (RBL)-2H3 cells. One of these compounds, (-)-cyclopenol, significantly decreased the degranulation release with an IC50 value of 60.3 μM, compared to that of 91.6 μM of the positive control, loratadine.

Ruiz-Domínguez et al. [21] studied the effect of two different drying methods for the recovery of lutein extracted by the supercritical fluid extraction process from the microalgae *Muriellopsis* sp (MCH35), which is isolated from an arid region of the north of Chile, Antofagasta, where it is exposed to high solar radiation. Among microorganisms as a prolific source of bioactive molecules, microalgae are the most diversified photosynthetic organisms with high adaptability to various environmental conditions [22]. Microalgae are rich with bioactive molecules with healthy benefits and have potential applications in pharmacological, nutraceutical, cosmeceutical, and biotechnological sectors. Lutein is a carotenoid belonging to the class of terpenoids, and due to its antioxidant potential, it displays a beneficial role to human health in ameliorate cardiovascular diseases [23], various types of cancer [24], and age-related macular degeneration [25]. Since several strategies have been studied to enhance microalgal carotenoid production, *Muriellopsis* sp. (MCH35) represents a potential candidate for lutein production under intense UV irradiation for biotechnological applications.

Wang et al. [26] reported the identification and characterization of a crustin from the shrimp *Rimicaris* sp. inhabiting the deep-sea hydrothermal vent in Manus Basin (Papua New Guinea). Shrimps belonging to the family Alvinocarididae are particularly abundant in the deep waters of Atlantic, Pacific, and Indian Oceans, especially in hydrothermal vents and cold seeps. Crustin, a cationic peptide of 7–22 kDa, belongs to the class of antimicrobial peptides (AMPs), a class of evolutionarily conserved molecules that exist in almost all organisms and play an important role in the innate immunity of organisms. They directly kill bacteria, fungi, viruses, and parasites, targeting the inner and/or outer membranes of microorganisms in a non-receptor-specific manner, with a rate of resistance several orders of magnitude lower than that of conventional antibiotics. 

As Guest Editor of the second edition of this Special Issue, I dedicate this Issue to the memory of Prof. Guido di Prisco, my mentor, who passed away in September 2019. He spent his life studying the structure, function, and evolution of the hemoglobins of polar fishes and inspired my interest in life in Antarctic and Arctic environments. I am also grateful to all the authors who contributed to making this an exceptional Special Issue with new discoveries/research on bioactive molecules from extreme conditions. The papers included in this second Special Issue, as well as in the first Special Issue, highlight the increasing interest in these extreme habitats, which are perceived as important sources for drug discovery. I want to thank all the reviewers and *Marine Drugs* for their support and kind help.

I hope that this collection will provide a unique and valuable reference source for researchers interested in extreme environments and help the scientific community inspire the next generation to further research dedicated to this field.

## Data Availability

Not applicable.
